# Reliable Deep Learning–Based Detection of Misplaced Chest Electrodes During Electrocardiogram Recording: Algorithm Development and Validation

**DOI:** 10.2196/25347

**Published:** 2021-04-16

**Authors:** Khaled Rjoob, Raymond Bond, Dewar Finlay, Victoria McGilligan, Stephen J Leslie, Ali Rababah, Aleeha Iftikhar, Daniel Guldenring, Charles Knoery, Anne McShane, Aaron Peace

**Affiliations:** 1 Faculty of Computing, Engineering & Built Environment Ulster University Jordanstown United Kingdom; 2 Faculty of Life & Health Sciences, Centre for Personalised Medicine Ulster University Londonderry United Kingdom; 3 Department of Diabetes & Cardiovascular Science University of the Highlands and Islands Inverness United Kingdom; 4 HS Kempten, Kempten, Germany Hochschule Kempten Kempten Germany; 5 Emergency Department Letterkenny University Hospital Donegal Ireland; 6 Western Health and Social Care Trust Londonderry United Kingdom

**Keywords:** deep learning, ECG interpretation, electrode misplacement, feature engineering, machine learning, ECG, engineering, cardiovascular disease, myocardial infarction, myocardial, physicians

## Abstract

**Background:**

A 12-lead electrocardiogram (ECG) is the most commonly used method to diagnose patients with cardiovascular diseases. However, there are a number of possible misinterpretations of the ECG that can be caused by several different factors, such as the misplacement of chest electrodes.

**Objective:**

The aim of this study is to build advanced algorithms to detect precordial (chest) electrode misplacement.

**Methods:**

In this study, we used traditional machine learning (ML) and deep learning (DL) to autodetect the misplacement of electrodes V1 and V2 using features from the resultant ECG. The algorithms were trained using data extracted from high-resolution body surface potential maps of patients who were diagnosed with myocardial infarction, diagnosed with left ventricular hypertrophy, or a normal ECG.

**Results:**

DL achieved the highest accuracy in this study for detecting V1 and V2 electrode misplacement, with an accuracy of 93.0% (95% CI 91.46-94.53) for misplacement in the second intercostal space. The performance of DL in the second intercostal space was benchmarked with physicians (n=11 and age 47.3 years, SD 15.5) who were experienced in reading ECGs (mean number of ECGs read in the past year 436.54, SD 397.9). Physicians were poor at recognizing chest electrode misplacement on the ECG and achieved a mean accuracy of 60% (95% CI 56.09-63.90), which was significantly poorer than that of DL (*P*<.001).

**Conclusions:**

DL provides the best performance for detecting chest electrode misplacement when compared with the ability of experienced physicians. DL and ML could be used to help flag ECGs that have been incorrectly recorded and flag that the data may be flawed, which could reduce the number of erroneous diagnoses.

## Introduction

### Background

Clinicians routinely face the challenge of making sense of a large amount of high-dimensional and heterogeneous data to inform their clinical decision making. Poor clinical decisions can fail to provide the correct diagnosis and treatment, which can have a large impact on patient safety and health care costs [1,2]. Artificial intelligence technologies such as deep learning (DL) and machine learning (ML) could play an important role in developing smarter clinical decision-making algorithms that can assist clinicians in making accurate diagnoses. To operationalize artificial intelligence in health care, interactions between data scientists and clinicians are essential to maximize the use of clinical data in the development of automated and predictive systems [2,3].

Cardiovascular diseases are heterogeneous and complex in nature, as they can be caused by a plethora of environmental, genetic, or behavioral factors. To diagnose a cardiac disease, the provision of incorrect data such as electrocardiogram (ECG) data can have a high impact on clinical decision making. A known error is an incorrectly recorded ECG caused by placing precordial electrodes (chest electrodes: V1, V2, V3, V4, V5, and V6) in incorrect positions, resulting in erroneous ECG signals that are interpreted by physicians to inform patient diagnostic signs and treatment plans. This is complicated by the fact that many physicians and cardiologists are not normally present when the ECG is being recorded by a nurse or ECG technician [4-7]. Therefore, ECG interpreters are unaware of the electrode positions that were used to record the ECG that they are reading. Electrode misplacement errors can affect the clinical interpretation of ECGs [8].

Research has shown that signals recorded by electrode V2 are very sensitive to misplacement, followed by electrodes V3, V1, and V4, whereas electrodes V5 and V6 have little visible changes in ECG morphology [9]. The most common error in electrode misplacement is placing electrodes V1 and V2 too high in the third or second intercostal space (ICS). The correct position of electrode V1 is in the fourth ICS at the right sternal edge and that of V2 is in the fourth ICS at the left sternal edge. Correctly placing the electrodes V1 and V2 is crucial, given that their misplacement is also known to cause subsequent misplacement of the remaining chest electrodes (V3 to V6) [9]. Electrode misplacement in ECG acquisition can occur between 40% and 60% of the time [10,11]. Approximately 50% of V1 and V2 electrodes are placed wide and high of their correct anatomical position [10,11].

According to one study, incorrect electrode placement could lead to incorrect diagnoses in 17% to 24% of patients [12]. ECG signals recorded from vertically misplaced V1 and V2 electrodes could also result in a false diagnosis of Brugada syndrome [13] and a failure to detect myocardial infarction (MI) and left ventricular hypertrophy (LVH) [10]. Misplacement can not only conceal but also *mimic* other cardiac diseases, such as MI [14-16]. Less than 20% of cardiologists and 50% of nurses can correctly place V1 and V2 in their correct positions [17]. Several devices have been devised and used to correctly place precordial electrodes. One of the technologies involves using a sliding ruler to facilitate the positioning of electrodes in the correct position [18]. Unfortunately, these technologies have not been widely adopted, likely because of an increase in cost.

To date, research in this area has focused on algorithm development to detect limb electrode interchanges [17-20] rather than precordial electrode misplacement, because the latter is more challenging. Schijvenaars et al [21] used body surface potential maps (BSPMs) to derive transformation matrices to mimic electrode misplacement errors; therefore, BSPMs are suitable for studying electrode misplacement errors.

### Objectives

The aim of this study is to determine the performance of ML and DL algorithms for detecting V1 and V2 electrode misplacement when recording ECGs (as V1 and V2 electrode misplacement can also result in misplacement of the other chest electrodes [V3-V6]) and to benchmark this performance against a group of physicians.

## Methods

### ECG Data Set Description

**Figure 1:**
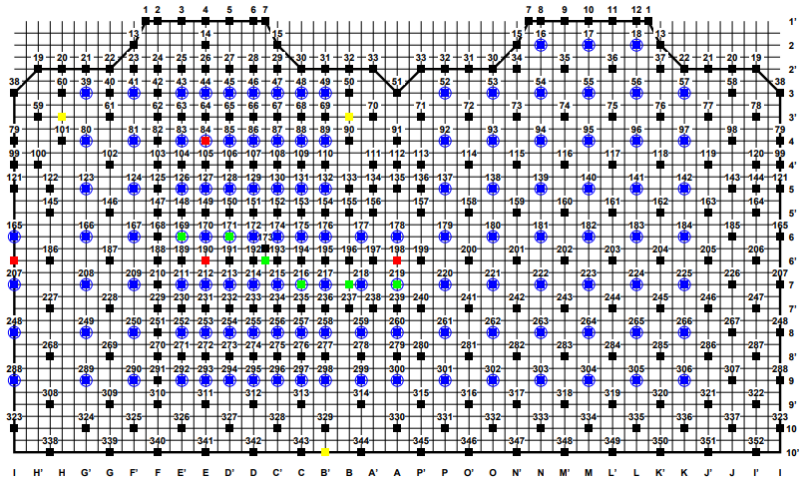
Body surface potential map. Symbols after letters represent column numbers, while symbols after numerals represent row numbers.

ECGs (V1 and V2 electrodes) were extracted from a high-resolution BSPM (Figure 1). Each BSPM comprises 117 nodes (ECG electrodes) and is known as the Kornreich data set [22-24]. This data set has been used in a large number of publications from groups around the world; however, no researcher has used it to train an algorithm to detect the misplacement of electrodes V1 and V2.

**Figure 2:**
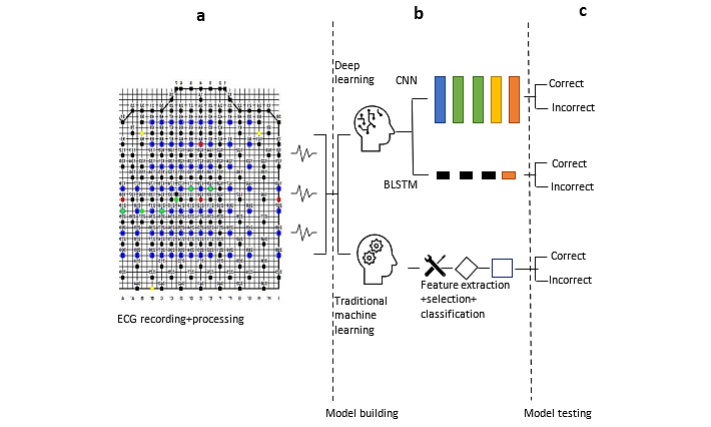
The data pipeline of this study using 3 phases (data engineering, analytics, and delivery). (a) The data engineering phase that includes data collection (extracting electrocardiograms from body surface potential maps) and data preparation (removing noise from extracted data). (b) The analytics phase that includes traditional machine learning (linear support vector machine, quadratic support vector machine, fine decision tree, coarse decision tree, logistic regression, and bagged tree) and deep learning (convolutional neural network and bidirectional long short-term memory network). (c) The delivery phase that is used to show traditional machine learning and deep learning model inferences. BLSTM: bidirectional long short-term memory; CNN: convolutional neural network; ECG: electrocardiogram.

The ECG data set consisted of three different subject types, including normal ECGs and ECGs showing MI and LVH. In this study, we have ECGs for 453 patients (normal: n=151, LVH: n=151, and MI: n=151). Each ECG was acquired at a sampling frequency of 300 Hz. For each BSPM, we extracted a correct ECG and an incorrect ECG (where electrodes V1 and V2 were misplaced). This provided a natural class balance where 50% of the cases are correct and 50% are incorrect. This is important given that algorithms improve their performance when being equally exposed to the same number of cases in each class so as to avoid bias and maximize *learning*. For preprocessing, the 117 nodes or electrodes in each BSPM are multiplied using a transformation matrix to obtain 352 nodes that provide greater resolution (using the Dalhousie torso [22], which is a standard approach). According to the recorded data set [25], nodes 169 and 171, denoted in green (Figure 1), represent electrodes V1 and V2, respectively, in their correct positions (fourth ICS). We used nodes 126 and 128, denoted in blue (Figure 1), to represent the misplaced electrodes V1 and V2 in the third ICS and nodes 83 and 85, in blue color, to represent V1 and V2 as misplaced in the second ICS. For each patient, we have recorded the ECG signals simultaneously for electrodes V1 and V2 and one cardiac cycle comprising PQRS deflections. Figure 2 shows an overview of the methodology used in this study.

### Detecting V1 and V2 Electrode Misplacement in the Second and Third ICSs Using Feature Engineering

Given that we have one ECG cycle for each patient, the signal was normalized using Equation 1 to reduce signal distortion and baseline drift.

*y* [n]=s[*n*]/max (|s[n]|) (1)

where s[n] is the input signal and y[n] is the output signal.

For feature extraction, a total of 16 ECG features were extracted using 3 different methods: (1) time-domain features (we considered 6 time-domain features, including P-wave amplitude, PR interval, QRS onset value, R-wave peak amplitude, offset of the QRS, and S-wave amplitude), (2) statistical domain features (including the mean, SD, skewness, and kurtosis of the ECG signal; Pearson correlation coefficient; and the root-mean-square error between V1 and V2 electrodes, given that these electrodes are commonly misplaced together), and (3) time-frequency features. The latter involved a discrete wavelet transform using 4 levels and a symlets mother wavelet function, 4 detailed coefficients (D1, D2, D3, and D4), and 4 approximation coefficients (A1, A2, A3, and A4). We also considered the maximum, minimum, and mean values of D4 as features.

For feature selection, a hybrid approach feature selection algorithm was used, which combined the filter and wrapper methods. A total of 16 features were ranked using different filter methods, including mutual information feature selection, maximum relevance minimum redundancy, joint mutual information (JMI), entropy, and relief. Second, a backward elimination algorithm was performed on ranked features to find an optimal set of features as inputs to the ML classifier. The backward elimination algorithm started with all 16 features and removed feature by feature until the best result was achieved.

For classification, we used 6 ML classifiers. This involved the use of three different types of decision trees (DTs): (1) fine DT, which is used to make many leaves that can enable the tree to make fine distinctions between classes; (2) a coarse DT (CDT) that is used to make a small number of leaves that can enable the tree to make coarse distinctions between classes; and (3) a bagged tree that uses bootstrapping with replacement to produce multiple training data sets and takes the majority outcome from multiple trees. Data will be presented using Equation 2.

(*X*;*Y*)=(*x_1_*,*x_2_*,... ..,*x_n_*; *Y*) (2)

where X represents features and Y represents classes.

Gini impurity (GI) was the splitting criterion used to split the tree into branches. In this study, there are two classes: (1) label 0 represents the incorrect electrode placement class and (2) label 1 represents the correct electrode placement class. Equation 3 is used to compute the GI for each class.

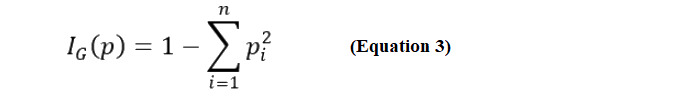



where n is the number of classes and pi is the fraction of subjects labeled with class i in the data set.

In addition to DT techniques, we used variants of the support vector machine (SVM) and logistic regression (LR). This includes a linear SVM that incorporates two parallel hyperplanes that are selected to separate the data set into two classes where the distance (margin) between hyperplanes should be as large as possible. Equations 4 and 5 describe the two hyperplanes.

*w.x*−*b*=1 (4)

*w.x*−*b*=−1 (5)

where w represents the weight corresponding to each feature, x features the data set, and b represents the biased term. Cases above this hyperplane or on the hyperplane should be in class 1, and cases below this hyperplane should be in class 0.

A quadratic SVM was used, where the quadratic kernel function was used to split the data set into two classes. The difference between linear SVM and quadratic SVM was the kernel function used to split the cases. Finally, LR or logit was used because this was a common statistical technique for binary classification. This technique used log odds (L) as computed using Equation 6, which represents a linear combination of features and model parameters.

L= α_0_ + α_1_.x_1_+...+ α_n_.x_n_ (6)

where α_0_ coefficients are model parameters and xn are features.

Odds (o) computation was the exponent that was used to compute odds using Equation 7, and the corresponding probability was computed using Equation 8.

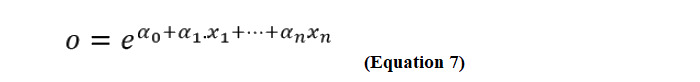

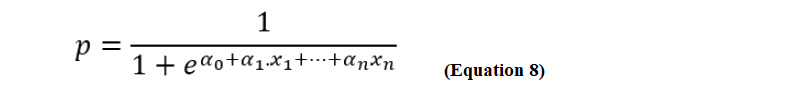





### Detecting V1 and V2 Electrode Misplacement in the Second and Third ICSs Without Feature Engineering

DL does not require feature engineering (ie, feature extraction and selection). Therefore, raw ECG signals are fed into a deep neural network without specifying features. DL can entail different types of networks and architectures. This study uses two different DL networks: (1) convolutional neural networks (CNNs) and (2) bidirectional long short-term memory (BLSTM) networks. A CNN has been built using 15 layers that comprise 1 input layer (used to feed in the ECG signals), 3 hidden convolutional layers (which uses a filter with a variable length to transform the input signal into a convolution layer), 3 batch normalization layers (used to normalize the output of a previous layer by subtracting the mean of batch and dividing this by the SD of the batch), 3 rectified linear unit layers (an activation function that is used to remove negative values), 2 max-pool layers (which combine the sequence output of the previous layer into one single value to reduce the number of parameters and computation in the network), 1 fully connected layer (which connects every neuron in one layer to every neuron in the next layer), 1 soft-max layer (which uses LR to generate probability for each class), and 1 final classification output layer. The BLSTM network comprises 1 sequence input layer, 2 BLSTM hidden layers (which are used to learn the network through each complete time series at each time step), 1 fully connected layer, 1 soft-max layer, and 1 classification output layer.

### Physician Detection of V1 and V2 Electrode Misplacements Using Visual Inspection of the ECG

A web-based survey including 30 random ECGs of V1 and V2 (ECGs of correct placement of V1 and V2 [n=15] and ECGs of incorrect placement of V1 and V2 [n=15]) was emailed to 20 participants at the International Society for Computerized Electrocardiology 2019 Conference and Computing in Cardiology 2019 Conference. Of the 20 participants, 11 responded to the survey. They were asked to classify V1 and V2 and whether they were placed correctly. In addition, they were asked about their age, employment status, and experience of reading an ECG (the number of ECGs they read in the past year). A total of 11 physicians responded to the web-based survey. Ethical approval was granted by the Faculty of Computing, Engineering, and Built Environment in Ulster University, Northern Ireland, United Kingdom.

## Results

### Feature Engineering

As mentioned earlier, 16 features were extracted using three different domains: (1) time domain, (2) statistical domain, and (3) time-frequency domain. Table 1 lists each feature ID along with the feature description. In feature selection (filter process), each feature selection algorithm sorts features from the highest priority feature to the lowest priority feature.

After feature selection, the 6 classifiers were applied, and the best classifier accuracy for detecting misplacement in the second ICS was a bagged DT, followed by CDT, fine DT, LR, quadratic SVM, and linear SVM.

**Table 1:** Feature IDs and descriptions.

Feature ID	Domain	Feature description
1	Time	P-wave amplitude
2	Time	PR interval
3	Time	QRS beginning value
4	Time	R amplitude
5	Time	End of QRS value
6	Time	S-wave amplitude
7	Statistical	Mean of ECG^a^ signal
8	Statistical	Variance of ECG
9	Statistical	SD of ECG signal
10	Statistical	Skewness of ECG
11	Statistical	Kurtosis of ECG signal
12	Time-frequency domain	Maximum value of D4^b^
13	Time-frequency domain	Minimum value of D4
14	Time-frequency domain	Mean value of D4
15	Statistical	Correlation coefficient between V1 and V2 ECGs
16	Statistical	Root-mean-square error between V1 and V2 ECGs

^a^ECG: electrocardiogram.

^b^D4: decomposition coefficient 4.

For detecting electrode misplacement in the third ICS, the best classifier accuracy was also a bagged DT, followed by CDT, LR, quadratic SVM, linear SVM, and fine DT. Table 2 shows the accuracy of each classifier corresponding to each feature selection algorithm. The numeric appended to the label of each feature selection algorithm shows the optimal number of features that was used to achieve the best accuracy. On the basis of classifier accuracy, the best feature selection algorithm performance was JMI for detecting misplacement in the second ICS and RELIEF and JMI for the third ICS.

**Table 2:** Accuracy of the feature engineering classifiers using machine learning.

Classifier	Percent accuracy in the second ICS^a^						Percent accuracy in the third ICS				
	Entropy15	JMI^b^15	MIFS^c^14	MRMR^d^13	RELIEF16	ENTROPY14		JMI16	MIFS14	MRMR15	RELIEF16
Fine tree	85	85	85	82	84	60		59	60	58	59
Coarse tree	87	87	87	85	87	69		69	69	69	69
Logistic	82	82	83	81	82	64		63	65	63	63
SVM^e^	78	78	75	76	78	59		60	61	61	60
Q-SVM^f^	79	79	78	79	79	58		60	62	60	60
Bagged	88	92	90	92	90	69		70	66	69	70

^a^ICS: intercostal space.

^b^JMI: joint mutual information.

^c^MIFS: mutual information feature selection.

^d^MRMR: maximum relevance minimum redundancy.

^e^SVM: support vector machine.

^f^Q-SVM: quadratic support vector machine.

Superscripts such as MIFS13 represent the best number of features to provide a good accuracy in feature selection algorithm.

### DL (Without Feature Engineering)

**Figure 3:**
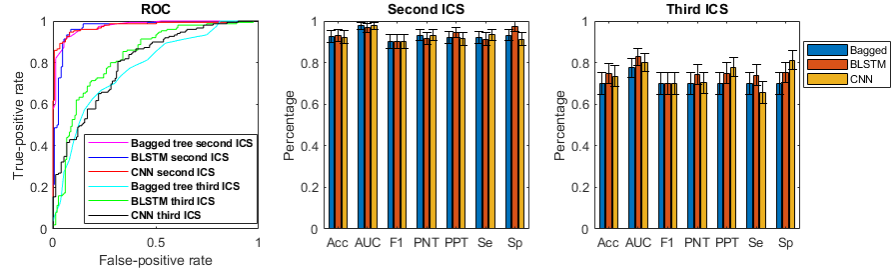
Receiver operating characteristic curves and other metrics results for deep learning and machine learning for detecting electrode misplacement in the second and third intercostal spaces. BLSTM: bidirectional long short-term memory; CNN: convolutional neural network; PNT: predictivity of negative test; PPT: predictivity of positive test.

As mentioned previously, two different DL networks were developed using different architectures. BLSTM achieved the best accuracy compared with the CNN (Table 3) and also outperformed the best accuracy achieved by the feature-engineered ML classifiers for detecting electrode misplacement in both the second and third ICSs. Figure 3 shows the accuracy, sensitivity, specificity, and receiver operating characteristic curve for BLSTM, CNN, and bagged tree in the second and third ICSs.

**Table 3:** Classification accuracy using two deep learning networks.

Classifier	Percent accuracy in the second ICS^a^	Percent accuracy in the third ICS
BLSTM^b^	93.0	74.7
CNN^c^	92.3	73.5

^a^ICS: intercostal space.

^b^BLSTM: bidirectional long short-term memory.

^c^CNN: convolutional neural network.

### Physicians Performance in the Second ICS

**Figure 4:**
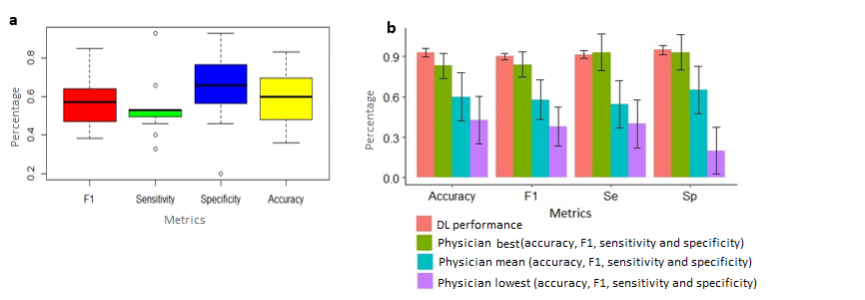
Physicians’ performance for classifying electrocardiograms as correctly recording or as recording with V1 and V2 misplacement in the second intercostal space: (a) physicians’ performance and (b) comparison of deep learning performance with physicians' performance regarding different metrics. Error bars were derived using 95% CI (constant=1.96); the pale red bars represent the deep learning performance, whereas the other colors (green, light blue, and purple) represent the physicians’ performance (best, mean, and lowest performance, respectively).

Performance of 11 physicians (age 47.3, SD 15.5) who were experienced in reading ECGs (mean number of ECGs interpreted in the past year 436.54, SD 397.9) were evaluated using F1 (mean 0.57, SD 0.14), sensitivity (mean 54.5%, SD 15), specificity (mean 65.4%, SD 21), and accuracy (mean 60%, SD 15) when detecting misplacement electrodes V1 and V2 in the second ICS (Figure 4). The accuracy achieved by DL was greater by a factor of 1.5, when compared with the average accuracy of physicians (*P*<.001).

## Discussion

### Principal Findings

On the basis of the medical literature review, ECG electrode misplacement is one of the most critical issues affecting ECG interpretation [8], especially given that it can cause misdiagnoses and inappropriate treatment and potentially a lack of appropriate treatment for the patient. The most common error in chest electrode placement is misplacing electrodes V1 and V2 too high from their correct position that can change ECG morphology and as a result cause a misdiagnosis. In this paper, we present new methods for detecting chest electrode misplacement using 2 approaches: (1) feature-engineered traditional ML algorithms and (2) DL (without any feature engineering) to detect V1 and V2 misplacement. This study describes the first experiment that uses DL to autodetect chest electrode misplacement, whereas previous work mainly focused on limb electrode interchanges. The BLSTM DL network achieved the highest performance in detecting V1 and V2 misplacement in the second ICS with an accuracy of 93.0% and in the third ICS with an accuracy of 74.7%. The ML algorithm (bagged tree) achieved a similar performance with an accuracy of 92.7% (for the second ICS detection) and 70.0% (for the third ICS detection). The performance of the bagged tree and the DL algorithms (BLSTM and CNN) are quite similar, whereas the performance of the other ML algorithms (F tree, C tree, LOG, SVM, and quadratic SVM) is statistically significantly different (*P*=.01) when compared with the performance of BLSTM, CNN, and bagged tree. A total of 11 medical doctors who were experienced in reading ECGs were recruited to detect electrode misplacement in the second ICS using the same data set to benchmark the ML and DL models. Furthermore, the physicians were biased as they were instructed to identify ECGs that appeared to be recorded incorrectly with respect to the V1 and V2 electrodes. On the basis of their performance, there was a significant difference (*P*<.001) when compared with the performance achieved by the ML and DL algorithms. Therefore, DL and ML can be used to help flag ECGs that have been incorrectly recorded and flag that the data may be flawed.

More generally, this study is particularly unique as many studies have focused on demonstrating the ability of DL to diagnose patients by automatically interpreting x-rays or ECGs, whereas this study focuses on using DL to detect medical errors. The use of DL to diagnose patients seems to be heavily criticized, given that DL lacks transparency and its decision logic cannot be easily explained to an end user. Therefore, DL for diagnostics elicits many trust issues and may not be widely adopted for this reason. However, physicians may accept black-box systems if they are being used for other subtasks, such as detecting errors, as opposed to providing a patient diagnosis.

### Limitations

This study has a number of limitations. The data set is limited and contains only three types of patients (those with MI, LVH, or normal sinus rhythm). Therefore, in further research, new types of patient cases need to be included to increase the data set size and to augment DL performance. Furthermore, the number of participants that manually detected correct or incorrect ECGs was small (n=11), with the limitation being that this cohort may not be a representative sample to benchmark with the ML algorithm. However, the results can be used as a direction for future investigations. The algorithms used were binary in nature and were not tested on many different types of misplacements and variations of ECG recordings. Therefore, a small random variation should be included for all chest electrodes (V1-V6). The performance of the presented algorithms in the real-world setting might not be as accurate as in the study because the algorithm would need to be prospectively tested with patient cases and with different data sets in diverse settings. Moreover, because the misplacement of V1 and V2 can also result in the misplacement of the remaining leads (V3-V6), there is also a need to further understand the impact of the misplacement of V3-V6 electrodes. The performance of the physicians in detecting the misplacement of V1 and V2 electrodes is likely to be lower in the real world as we instructed the subjects to *look out for* and detect the misplacement of V1 and V2 electrodes, which is not likely a condition or a high priority that is at the forefront of a physician’s mind when reading an ECG in clinical practice. Given that the ML features used to detect V1 and V2 are somewhat generic, this feature set could be reduced or refined by further clinical insight from experts and the literature that detail V1 and V2 signal morphology when misplaced.

### Conclusions

Implementing the algorithms invented in this study could improve ECG data quality, which can, in turn, improve decision making in cardiac care. We can conclude that DL provides the best performance for detecting chest electrode misplacement when compared with ML-based models and the ability of experienced physicians. Therefore, the medical device industry should consider DL to detect chest electrode misplacement. The results clearly show that the greater the misplacement (ie, in the second ICS), the greater the model accuracy. Therefore, in our future research, we aim to improve the accuracy of detecting chest electrode misplacement in the third ICS using alternative techniques rSr’ prime. However, adopting these algorithms in health care will take time and will be expensive as it may require prospective testing as part of a trial and approval from different regulatory organizations such as the Food and Drug Administration. However, given that this algorithm is used to flag potential errors and does not provide a diagnosis or recommend treatment, the risks are perhaps less severe. There may still be other costs, including staff training and integrating the algorithm into ECG machines. Future work will also involve the generation of saliency maps that can be used to explain how the DL algorithm is making its decision. This will facilitate knowledge discovery and may result in new ECG features that are characteristic of electrode misplacement.

## Abbreviations

BLSTM: bidirectional long short-term memory
BSPM: body surface potential map
CDT: coarse decision tree
CNN: convolutional neural network
DL: deep learning
DT: decision tree
ECG: electrocardiogram
GI: Gini impurity
ICS: intercostal space
JMI: joint mutual information
LR: logistic regression
LVH: left ventricular hypertrophy
MI: myocardial infarction
ML: machine learning
SEUPB: Special EU Programmes Body
SVM: support vector machine
